# Long non-coding RNA *NEAT1* promotes bone metastasis of prostate cancer through N6-methyladenosine

**DOI:** 10.1186/s12943-020-01293-4

**Published:** 2020-12-12

**Authors:** Simeng Wen, Yulei Wei, Chong Zen, Wei Xiong, Yuanjie Niu, Yu Zhao

**Affiliations:** 1 Department of Urology, The Second Hospital of Tianjin Medical University, Tianjin Medical University, Tianjin, 300211 China; 2 Department of Gynecology and Obstetrics, Tianjin First Central Hospital, Tianjin, 300192 China; 3 Department of Urology, Central South University, Changsha, 410011 China; 4 Department of Biochemistry and Molecular Biology, Mayo Clinic, Rochester, MN 55905 USA

**Keywords:** Bone metastatic prostate cancer, m6A, *NEAT1–1*, CYCLINL1, CDK19, ncRNA

## Abstract

**Background:**

N6-methyladenosine (m6A) is the most prevalent messenger RNA modification in mammalian cells. However, the disease relevant function of m6A on specific oncogenic long non-coding RNAs (ncRNAs) is not well understood.

**Methods:**

We analyzed the m6A status using patients samples and bone metastatic PDXs. Through m6A high-throughput sequencing, we identified the m6A sites on *NEAT1–1* in prostate bone metastatic PDXs. Mass spec assay showed interaction among *NEAT1–1*, CYCLINL1 and CDK19. RNA EMSA, RNA pull-down, mutagenesis, CLIP, western blot, ChIP and ChIRP assays were used to investigate the molecular mechanisms underlying the functions of m6A on *NEAT1–1*. Loss-of function and rescued experiments were executed to detect the biological roles of m6A on *NEAT1–1* in the PDX cell phenotypes in vivo.

**Results:**

In this study, we identified 4 credible m6A sites on long ncRNA *NEAT1–1.* High m6A level of *NEAT1–1* was related to bone metastasis of prostate cancer and m6A level of *NEAT1–1* was a powerful predictor of eventual death. Transcribed *NEAT1–1* served as a bridge to facility the binding between CYCLINL1 and CDK19 and promoted the Pol II ser2 phosphorylation. Importantly, depletion of *NEAT1–1*or decreased m6A of *NEAT1–1* impaired Pol II Ser-2p level in the promoter of *RUNX2*. Overexpression of *NEAT1–1* induced cancer cell metastasis to lung and bone; xenograft growth and shortened the survival of mice, but *NEAT1–1* with m6A site mutation failed to do these.

**Conclusion:**

Collectively, the findings indicate that m6A on ncRNA *NEAT1–1* takes critical role in regulating Pol II ser2 phosphorylation and may be novel specific target for bone metastasis cancer therapy and diagnosis. New complex CYCLINL1/CDK19/*NEAT1–1* might provide new insight into the potential mechanism of the pathogenesis and development of bone metastatic prostate cancer.

**Supplementary Information:**

The online version contains supplementary material available at 10.1186/s12943-020-01293-4.

## Introduction

Prostate cancer is the most common malignancy among males in the world, and also is the second-most common cause of cancer death in United States (US) males [1, 2]. Metastasis is main cause of death in prostate cancer. Prostate cancer has an affinity to metastasize to bone, about 80% of the metastatic prostate cancer cells spread to bones, such as the hip, spine, and pelvis bones [3, 4]. With androgen sensitive advanced prostate cancer, approximately 50% of men will develop bone metastases within two years [5]. 56% of prostate cancer patients without bone metastases were alive at five years, compared to 3% of patients with bone metastases. It suggests that bone metastatic prostate cancer is a poor prognosis in men and correlates with significant mortality [6]. Prostate cancer invades or travels through your blood or lymphatic system and once relocated, the cells begin to grow as “new” tumors. The molecular event for bone metastatic tumor remains unclear.

Over 100 types of cellular RNA modifications, N6-methyladenosine (m6A) is the most abundant post-transcriptional RNA modification on eukaryote RNAs [7–9]. The functions of m6A in mRNA include nuclear transport, splicing, stability and translation [10–14]. However, the m6A function in long non-coding RNA is limit to report. The nuclear-enriched abundant transcript 1(*NEAT1*) is an overexpressed long non-coding RNA in many human cancer types [15]. The higher expression of *NEAT1* is significantly associated with worse survival in cancer patients [15, 16]. *NEAT1* is responsible for several cancer clinical features: patient survival, metastasis, recurrence, stem cell-like phenotype [16]. Knockdown of *NEAT1* can decrease the malignant behavior of tumor cells for breast cancer [17, 18], hepatocellular carcinoma [19], laryngeal squamous cell carcinoma [20], lung cancer [21], glioma [22], prostate cancer [23], and skin cancer [24]. *NEAT1* is identified as an essential component of nuclear paraspeckles [25] and mediates transcription [26, 27]. *NEAT1* is transcribed from familial tumor syndrome multiple endocrine neoplasia type I loci and encodes two transcriptional variants, *NEAT1–1* (~ 3.7 kb) and *NEAT1–2* (~ 22.7 kb) [28]. *NEAT1–1* serves as the ERα-inducible oncogenic target and promotes the cancer progression in castrate-resistant prostate cancer (CRPC) [23]. On the contrary, *NEAT1–1* can be regulated by p53 to suppress the cancer transformation in pancreatic cancer [29, 30].

In this study, we identified the novel m6A function in ncRNA that played an oncogenic role in bone metastatic prostate cancer and was correlated with poor prognosis. Further experiments demonstrated that *NEAT1–1* m6A facilitated the oncogenic function of new complex CYCLINL1/CDK19 for Pol II Ser2 phosphorylation. The regulatory network involving the new complex CYCLINL1/CDK19/*NEAT1–1* might provide new insight into the potential mechanism of the pathogenesis and development of bone metastatic prostate cancer.

## Methods

### Plasmids, reagents and antibodies

GST-tagged Pol II-C terminal were generated by cloning the corresponding cDNAs into pGEX-4 T-1 vector. The cDNA fragments were amplified by Phusion polymerase (NEB, USA) using Phusion High-Fidelity PCR Master Mix. pCRII-NEAT1–1 was kind gift from Dr. Xiong in Second Xiangya Hospital [31]. Mammalian expression plasmids for CYCLINL1, CDK19 and *NEAT1–1* were sub-generated by inserting each target cDNA into the pcDNA3.1 vector. The insert and deletion mutants were constructed using QuikChange II Site directed-Mutagenesis Kit (Agilent, USA). Antibodies and primers were shown in Table S1.

### Cell lines, cell culture and reagents

Short term explant cultures were obtained from a primary patient derived bone metastatic prostate panel. PDXs related primary cells were cultured in RPMI 1640 medium supplemented with 10% FBS or charcoal-stripped fetal bovine serum (FBS) (Invitrogen) (androgen-depleted medium) and 100 μg/ml penicillin-streptomycin-glutamine (Invitrogen) at 37 °C with 5% CO_2_. Cell authentication was performed using STR profiling as previously description [32, 33].

### Human prostate cancer specimens and RNA isolation from human tissues

Formalin-fixed paraffin-embedded (FFPE) or fresh-frozen hormone-naïve primary prostate cancer and bone metastatic tissues were randomly selected from the Tianjin Medical University Tissue Registry and Second Xiangya Hospital. Hormone-naïve patients with biopsy-proven prostate cancer have been treated at Tianjin Medical University by radical retropubic prostatectomy between January 1995 and December 2018 without neoadjuvant therapy. The clinical tissues were approved by the Tianjin Medical University Institutional Review Board and Medical Ethics Committee. FFPE tissues were collected and total RNAs were isolated using a RecoverAll Total Nucleic Acid Isolation Kit (Life Technologies). Isolation of RNAs from frozen human prostate cancer tissues was performed as described previously [34].

### Patient-derived xenograft (PDX)

PDXs were established through injection of surgical tissues into the flank of male athymic nude mice 6–8 weeks of age. The procedures for establishment and maintenance of flank xenografts and short-term explant cultures were followed the published procedures from Mayo Clinic Patient-Derived Xenograft National Resource Center [35]. P-18 is the Number 18 of 66 PDXs in Tianjin Medical University, and is the orthotopic metastasis prostate cancer tissue from a 76 years old patient’s right pelvis after a surgery. P-34 is the Number 34 of 66 PDXs in Tianjin Medical University, and is the orthotopic metastasis prostate cancer tissue from an 86 years old patient after a surgery. Mice were housed in the Tianjin Medical University pathogen-free rodent facility. All procedures were approved by the Tianjin Medical University Institutional Animal Care and Use Committee.

### Mouse xenograft generation and tumor growth measurement

The mouse study was approved by Tianjin Medical University Institutional Animal Care and Use Committee. Six-week- old NSG male mice were injected with 5 × 10^6^ of cancer cells infected with lentivirus or shRNAs and/or expression vectors in 100 μl PBS with 100 μl of Matrigel matrix (BD Bioscience) in right flanks. After injection of tumor cells into mice, tumors were monitored until they reach maximum tumor volumes of 1000 mm^3^ and tumor growth was measured with caliper every 6 days.

### Mouse tail-vein injection of tumor cells and bioluminescent imaging

The mouse study was approved by Tianjin Medical University Institutional Animal Care and Use Committee. Male NSG mice were used for the experiment. P-18 cells stably expressing the luciferase genes were infected with lentivirus for empty vector or *NEAT1–1* WT and m6A-#4 site mutants. 1 × 10^6^ infected cells in 0.2 ml PBS were injected via tail vein into individual mice (six mice each group). Mice were monitored by bioluminescent imaging. Mice were injected with luciferin (300 mg/ kg) 10 min before imaging. Mice were anaesthetized (3% isoflurane) and imaged using the IVIS spectrum imaging system (Xenogen, Life Sciences). Images were analyzed with Living Image software (Xenogen, Life Sciences). Bioluminescent flux (photons/s/sr/cm^2^) was determined for lesions in lung and body.

### In vitro transcription and RNA pulldown by GST recombinant proteins

A fragment or full length, corresponding to the *NEAT1–1* RNA, was subcloned into the pcDNA3.1 backbone vector. Mutations with various deletions within this region were generated by mutagenesis using a KOD-Plus-Mutagenesis Kit (TOYOBO). Myc-CYCLINL1 and Flag-CDK19 were translation using TNT Quick coupled transcription/translation system kit (Promege). Wild-type and mutated vectors were linearized by digestion with XhoI and purified by the Gel Extraction Kit (Qiagen). 200 ng linearized plasmid DNAs were transcribed in vitro using T7 RNA polymerase. RNAs were treated with DNase I to eliminate the template DNA. GST-Pol II-C terminal recombinant proteins were expressed in *E. coli* (BL21) after induction with 0.5 mM IPTG at 16 °C for 12 h and purified with glutathione Sepharose 4B beads (GE Healthcare) as described previously [34]. Purified GST recombinant proteins were incubated with in vitro transcribed RNA in RNA structure buffer (50 mM Tris, pH 7.4, 150 mM NaCl; 1 mM MgCl_2_) [34]. For the GST-Pol II-C binding assay, ATP and MgCl_2_ was added to the binding buffer to a final concentration of 1 mM. After extensive washes, RNAs were purified using the RNeasy MinElute Cleanup Kit (Qiagen). Precipitated RNA was detected by real-time RT-PCR.

### Design and screening of highly optimized generation-2.5 antisense oligonucleotides (ASOs)

The ASOs used in this study contained a full phosphorothioate backbone and a 10-base 2′-deoxynucleoside gap flanked by 2′-O-methyl (cMt)-modified nucleotides. The motif for the ASOs targeting the *NEAT1–1* RNA tested was mmm-10-mmm, where m represents cMt modification and − 10- represents the 10-base DNA gap. ASOs were synthesized and purified as described previously [36]. A large number of ASOs targeting sense-strand *NEAT1–1* RNA were screened by Rebio Pharmaceuticals Inc. (Guangzhou, China) for high efficient reduction of RNA. ASO1 (5′- GAGTGATGTGGAGTTA-3′) and ASO2 (5′- GGCTCTTCTGGATTTG-3′) that resulted in strong reduction of *NEAT1–1* RNA were selected for further studies. Negative control ASO (5′-GGCTACTACGCCGTCA-3′) with the same chemistry, but matching no human transcripts, Control were included in each experiment to demonstrate the specificity of *NEAT1–1* ASOs. These oligonucleotides were designed to exclude G-strings with four Gs or two sets of three Gs in a row to prevent non-antisense-mediated effects [34].

### RNA isolation from cultured cells, reverse transcription PCR (RT-PCR) and real-time PCR

Total RNA was isolated from cultured cells using TRIzol reagent (Invitrogen) or the RNeasy Plus Mini Kit (Qiagen) according to the manufacturer’s instructions. First-strand cDNA was synthesized with the PrimeScript Reverse Transcriptase Kit (TaKaRa Bio). Reverse transcription and real-time PCR were performed as described previously [37]. The PCR primers for AR new target genes are listed in Table S1.

### Biotin-labeled RNA pulldown and western blot analysis

RNAs were biotin-labeled during in vitro transcription using Biotin RNA Labeling Mix (Roche) and T7 polymerase (New England Biolabs). P-18 primary cells cultured in androgen-depleted medium were lysed in modified Binding buffer (50 mM Tris-HCl pH 7.5, 150 mM NaCl, 1% NP-40, 0.1% SDS and 1% protease inhibitor cocktails). Cell lysates were incubated with biotin-labelled RNAs and streptavidin beads at 4 °C for 12 h. The beads were washed in wash buffer (50 mM Tris, pH 7.4; 150 mM NaCl; 0.05% Nonidet P-40 (NP-40); 1 mM MgCl2) at 4 °C six times. The samples were subjected to western blot analyses as described previously [34]. Briefly, protein samples were denatured and subjected to SDS-polyacrylamide gel electrophoresis (SDS/PAGE), and were transferred to nitrocellulose membranes (Bio-Rad). The membranes were immunoblotted with specific primary antibodies, horseradish peroxidase-conjugated secondary antibodies, and visualized by SuperSignal West Pico Stable Peroxide Solution (Thermo Scientific). The antibodies are shown in Table S1.

### Chromatin immunoprecipitation (ChIP)

ChIP was performed as described previously [34]. The online Biosearch Technologies’ Stellaris FISH Probe Designer was used to design antisense oligo probes tiling *NEAT1–1* RNA. The probe oligos were synthesized with a 3′-Biotin-TEG modification and purified by HPLC.

### m6A RIP-seq and data analysis

The m6A RIP-seq data were analyzed from GSE63753 [38]. Pre-analysis quality control was performed using FastQC (http://www.bioinformatics.babraham.ac.uk/projects/fastqc/) and RSeQC software [39] to ensure that raw data are in excellent condition and suitable for downstream analyses. Pair-end raw reads were aligned to the human reference genome (GRch37/hg19) using Tophat [40]. Genome-wide coverage signals were represented in BigWig format to facilitate convenient visualization using the UCSC genome browser. Gene expression was measured using RPKM (Reads Per Kilo-base exon per Million mapped reads) as described previously [40]. Correlation analyses between RNA expressions were performed using Python and R scripts.

### RNA-seq and data analysis

P18 cells or P18-NEAT1–1 KO cells were transfected with ASO or NEAT1–1 plasmids for 48 h. Total RNAs were isolated from cells using the methods as described previously [34]. Briefly, RNA was isolated using RNeasy Plus Mini Kit (Qiagen). High quality total RNAs (Agilent Bioanalyzer RIN > 7.0) were employed for the preparation of sequencing libraries using Illumina TruSeq Stranded Total RNA/Ribo-Zero Sample Prep Kit. A total of 500–1000 ng of riboRNA-depleted total RNA was fragmented by RNase III treatment at 37^o^ C for 20 min and RNase III was inactivated at 65^o^ C for 10 min. Size selection (50 to 150 bp fragments) was performed using the FlashPAGE denaturing PAGE-fractionator (Life Technologies) prior to ethanol precipitation overnight. The resulting RNA was directionally ligated, reverse-transcribed and RNase H treated. Samples with biological duplicates were sequenced using the Illumina HiSeq2000 platform at the BGI Genomics (Shenzhen China). Pre-analysis quality control was performed using FastQC and RSeQC software to ensure that raw data are in excellent condition and suitable for downstream analyses. Pair-end raw reads were aligned to the human reference genome (GRch37/hg38) using Tophat. Genome-wide coverage signals were represented in BigWig format to facilitate convenient visualization using the UCSC genome browser. Gene expression was measured using RPKM (Reads Per Kilo-base exon per Million mapped reads). Correlation analyses between mRNA expressions in different groups were performed using Python and R scripts. EdgeR was used to identify genes that were differentially expressed between *NEAT1–1* knocking down or m6A-deletion.

### Chromatin immunoprecipitation (ChIP) and chromatin isolation by RNA purification (ChIRP)

ChIP was performed as described previously [34]. The ChIRP experiment was performed essentially as per the original protocol as described previously [34]. The online Biosearch Technologies’ Stellaris FISH Probe Designer was used to design antisense oligo probes tiling *NEAT1–1* RNA. The probe oligoes were synthesized with a 3′-Biotin-TEG modification and purified by HPLC. Two pools of probes were prepared, one with 10 μM of each even numbered probe (2, 4, 6, etc.) and the other with 10 μM of each odd numbered probe (1, 3, 5, etc.).The ChIP primers and ChIRP probes are shown in Table S1.

### Clustered regularly interspaced short palindromic repeats (CRISPR)-Cas9 system

CRISPR-Cas9 assay was performed using CRISPR-Cas9 tool kit (Santa Cruz). gRNAs were cloned into lentivirusV2 plasmid, under U6 rpomoter. The gRNAs are shown in Table S1.

### RNA electrophoretic mobility shift assay (RNA EMSA)

Biotin-labeled RNA probes were generated by in vitro transcription using cDNA containing T7 promoter and the *NEAT1–1* fragment RNA were purchased from BGI Genomics (Shenzhen, China). For the RNA EMSA assay, recombinant Myc-CYCLINL1 or Flag-CDK19, 100 ng/ml tRNA, and 1 lμg of biotin-labeled RNA probe were mixed in binding buffer (10 mM Tris–Cl, pH 7.5, 25 mM KCl, 10 mM MgCl2, 1 mM DTT) for 30 min at 25 °C. RNA–protein complexes were digested by adding 100 units of RNase-T1 for 15 min at 37 °C and then sepa- rated in 6% of native poly acrylamide gel. RNA–protein complexes were blotted with HRP-conjugated streptavidin and the final results were visualized by autoradiography.

### Co-immunoprecipitation (co-IP) and Western blot analysis

To immunoprecipitate the ectopically expressed Flag or Myc-tagged proteins, transfected cells were lysed 24 h post-transfection in BC100 buffer. The whole-cell lysates were immunoprecipitated with the monoclonal anti-Flag or anti-Myc antibody-conjugated agarose beads (Sigma-Aldrich) at 4 °C overnight. After three washes with lysis buffer, followed by two washes with BC100 buffer, the bound proteins were eluted using Myc or Flag-Peptide (Sigma-Aldrich) prepared in BC100 for 3 h at 4 °C. The eluted protein sample was resolved by SDS–PAGE. Cells cultured in androgen-depleted medium were lysed in modified Binding buffer (50 mM Tris-HCl pH 7.5, 150 mM NaCl, 1% NP-40, 0.1% SDS and 1% protease inhibitor cocktails). Cell lysates were incubated with biotin-labelled RNAs and streptavidin beads at 4 °C for 12 h. The beads were washed in wash buffer (50 mM Tris, pH 7.4; 150 mM NaCl; 0.05% Nonidet P-40 (NP-40); 1 mM MgCl2) at 4 °C six times. Briefly, protein samples were denatured and subjected to SDS-polyacrylamide gel electrophoresis (SDS/PAGE), and were transferred to nitrocellulose membranes (Bio-Rad). The membranes were immunoblotted with specific primary antibodies, horseradish peroxidase-conjugated secondary antibodies, and visualized by SuperSignal West Pico Stable Peroxide Solution (Fisher). The antibodies are shown in Table S1.

### Pol II CTD kinase assay

GST and GST-Pol II-C terminal domain (GST-Pol II-C, 372 amino acids of the Pol II-C terminal domain) recombinant proteins were incubated with CDK19 and CYCLINL1 translation products for kinase assay using the method as described previously [34]. The kinase reaction mixtures were incubated at 30 °C for 60 min and then stopped by the addition of SDS-PAGE buffer. The samples were measured using western blotting.

### RNA dot blot hybridization

The dot blot assays were performed as described previously [34, 37]. Briefly, equal amounts of biotin-labelled RNA were dot blotted and the blot was incubated with the Streptavidin-HRP antibody for 2 h and visualized by SuperSignal West Pico Stable Peroxide Solution (Thermo Scientific).

### RNA fluorescence in situ (FISH)

RNA FISH and imaging were performed as described previousely [41]. The probes targeting NEAT1–1, plasmids, and AR were designed by probe design software online (https://www.biosearchtech.com/stellaris-designer) and synthesized by BGI Genomics (Shenzhen, China). The probes were list in Table S1.

### Cross-linking immunoprecipitation (CLIP) and individual-nucleotide resolution CLIP (iCLIP)

5 × 10^6^ cells treated with 100 μM 4-Thiouridine (4SU) for 8 h were washed with cold PBS one time and cells were irradiated once with 150 mJ/cm2 at 365 nm using a Stratalinker. Cells were lysed in lysis buffer (50 mM Tris-HCL, pH 7.4, 100 mM NaCl, 1% NP-40, 0.1% SDS, 0.5% sodium deoxycholate, protease inhibitor cooktail and RNase inhibitors) with protease inhibitors (1 mL) and transferred to 1.5 mL microtubes. Lysate was partially digested by 2 U/μL RNaseT1/A for 15 min at 22 °C for iCLIP. RNA was immunoprecipitated with CYCLINL1 or CDK19 antibodies and protein A/G beads for 4 h at 4 °C. After washed for 4 times, RNA was phosphorylated by T4 PNK and ligated RNA between 3′ and 5′ ends by RNA T4 ligase. SDS-PAGE loading buffer was added and the mixture was incubated at 70 °C for 10 min. After running the SDS-PAGE gel, the RNA-protein complexes were transferred from gel to a nitrocellulose membrane using a wet transfer apparatus (30 V for 1 h). The membrane with target protein was cut up, and the targeted membrane piece was incubated with Proteinase K for de-crosslink. After de-crosslink, RNA was reverse transcribed into cDNA and subjected to real-time qPCR analysis.

### Statistical analysis

For in vivo experiments, animals were randomized. Randomization was not performed for all other experiments. All of the experiments were performed in biological triplicate unless otherwise specified. Statistical analyses were performed using R Script. Statistical analyses were performed using Student’s t test, or two-way ANOVA tests as indicated. *P* < 0.05 is considered statistically significant. Non-parametric Kolmogorov-Smirnov (KS) test was used to evaluate statistical significance of differential expression between primary prostate cancer and bone metastatic prostate cancer. Details regarding the statistical methods employed during RNA-seq and m6A RIP-seq data analyses were mentioned in aforementioned methods.

## Results

### m6A of *NEAT1–1* is elevated in prostate cancer and is a negative prognostic factor for patients

**Fig. 1:**
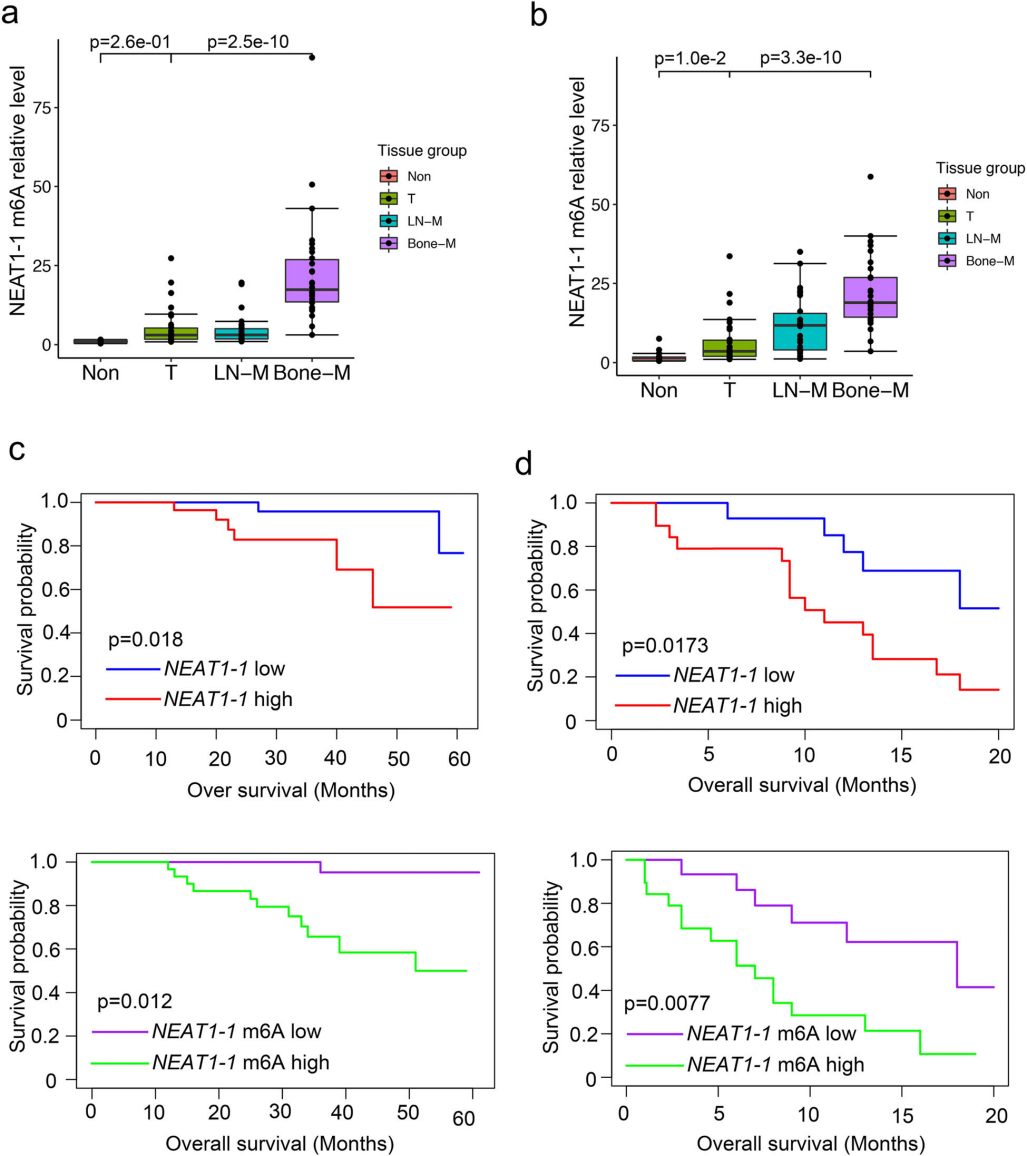
m6A of *NEAT1–1* was elevated in prostate cancer and was a negative prognostic factor for patients. **a** and **b** Box and whisker plot showing m6A signals upregulated in bone metastatic prostate cancer tissues. Analyswas of Second Xiangya Hospital (**a**) and Tianjin Medical University (**b**) data sets for levels of m6A and *NEAT1–1* RNA were based on the m6A-RIP and RT-PCR. *n* = 30 each group. *P* values were shown in the figures. **c** and **d** Kaplan-Meier survival analyswas of the Tianjin Medical University data sets for the relationship between the levels of m6A of *NEAT1–1*, expression of *NEAT1–1* and survival time in prostate cancers (**c**) and bone metastatic prostate cancer tissues (**d**). c, n = 30; d, *n* = 20. *P* values were shown in the figures

*NEAT1–1* as an oncogenic ncRNA is highly expression in multiple cancers. To understand the role of *NEAT1–1* in prostate cancer, we queried The Cancer Genome Atlas (TCGA; http://www.cbioportal .org) datasets. In the dataset, *NEAT1–1* expression was significantly higher in prostate cancer compare to normal tissues (Fig. S1a). Furthermore, elevated expression of *NEAT1–1* predicts poor patient prognosis (Fig. S1b). These data were consistent with pervious reported [23]. To further study the m6A modification of *NEAT1–1* in a clinicopathologically relevant context, we harvest the RNA immunoprecipitated by m6A antibody from fresh sample of adjacent normal cancer, primary cancer, lymph node-metastasis cancer and bone-metastatic cancer. Box plot analysis showed that m6A level of *NEAT1–1* was significantly higher in bone metastatic prostate cancer samples than primary prostate cancer or adjacent normal samples in two independent data sets with FFPE samples (Fig. 1a and b) and data set with frozen samples (Fig. S1c). Table 1 showed that m6A levels of *NEAT1–1* were statistically significant positive correlations with tumor metastasis and prostate specific antigen (PSA) recruitment, but not with the age, tumor stage and Gleason score. To further substantiate the survival significance of m6A on *NEAT1–1*, we analyzed the *NEAT1–1* expression, m6A level of *NEAT1–1* and its correlations with clinical behaviors of prostate cancer patients in our bone-metastasis cohort. Kaplan-Meier survival analysis of Tianjin Medical University data sets showed that high levels of *NEAT1–1* or m6A on *NEAT1–1* were associated with a shorter survival in prostate cancer patients with bone metastasis and overall survival in primary prostate cancer patients (Fig. 1c and d). Collectively, there was a correlation between *NEAT1–1* expression/m6A status and overall survival.

**Table 1:** The expressions of m6A on NEAT1 with clinic pathologic features

Characteristic		m6A level on NEAT1		*P*-value	
		High	low	High	low
Age	< 50	15	7	0.315	0.276
	≥50	30	8		
Gleason score	≤6	19	9	0.082	0.265
	> 6	26	6		
Tumor stage	I + II	22	6	0.063	0.226
	III + IV	23	9		
PSA recrutement (30	No	12	12	0.005	0.006
months)	yes	33	3		
metastasis	No	10	13	0.001	0.001
	yes	35	2		

### Four m6A sites were identified in *NEAT1–1* in prostate cancer

**Fig. 2:**
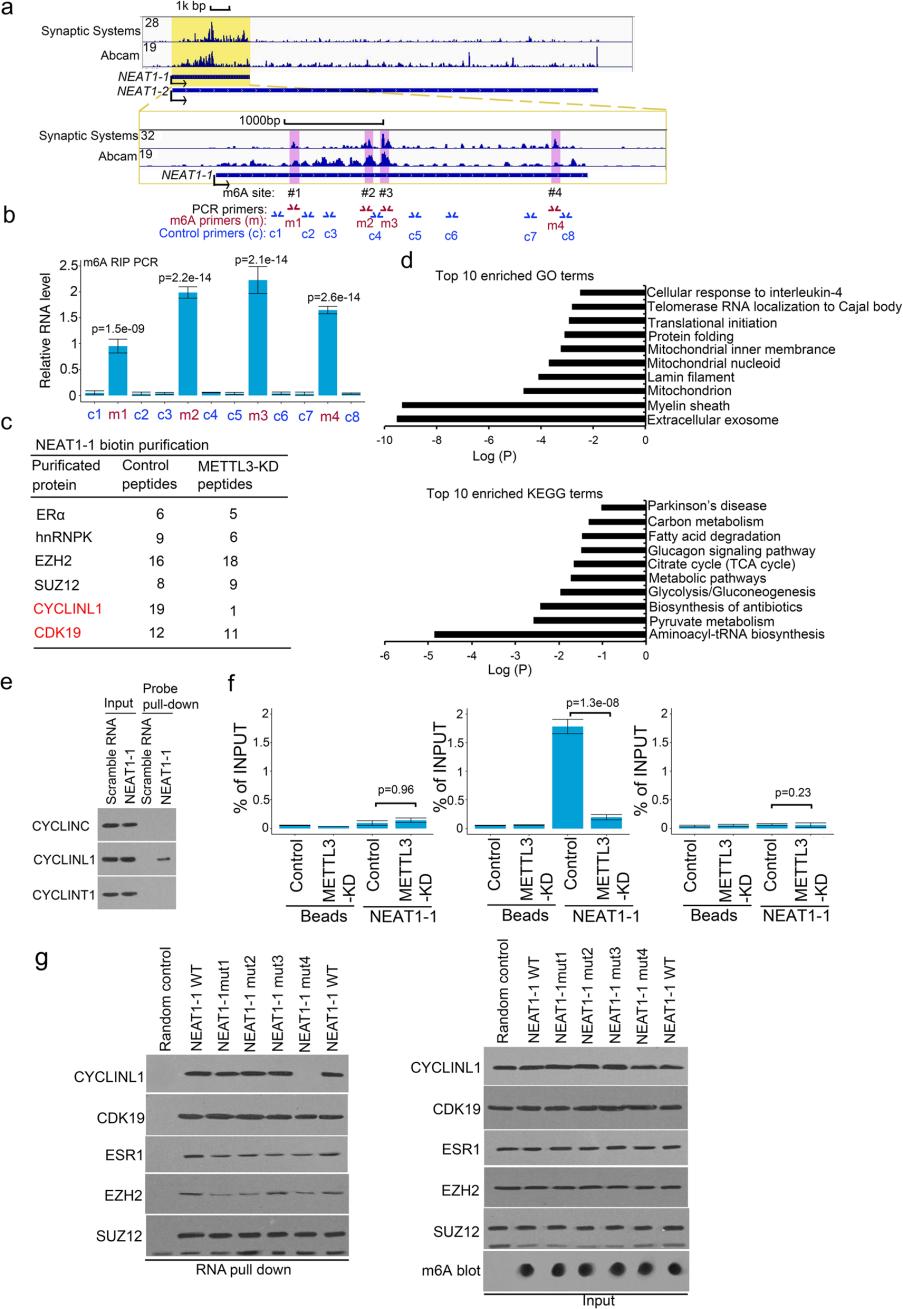
m6A sites were identified in *NEAT1–1* in prostate cancer. **a** m6A RIP-seq analyswas of m6A sites of *NEAT1* by two independent antibodies. The m6A profiles of *NEAT1* were shown in genome browser. **b** Quantitative PCR verification of m6A RIP results on the gene body of *NEAT1–1*with m6A antibody against the m6A sites in P-18 primary cells. Means and standard deviations (error bar) were determined from three replicates. Error bars represent mean ± SD for triplicate experiments. *P* values were shown in the figures. **c** The top hits of *NEAT1–1* pull-down proteins were identified by TAP-MS. The numbers of peptides were indicated in the lwast. **d** Ontology analyswas were by DAVID (david.ncifcrf.gov) and KEGG analyswas by DAVID and KAAS (www.genome.jp/kass-bin). **e** Biotin pull-down assay by incubating biotin-labeled specific probes targeting *NEAT1–1* with P-18 cell lysate followed by Western blot with CYCLINC1, CYCLINL1 and CYCLINT1 antibodies. **f** CLIP-qPCR analyswas of CYCLINL1 binding at the *NEAT1–1* in P-18 control or METTL3 knocking out cells transfected with control or METTL3-specific sgRNA. Immunoprecipitated RNAs were detected by real-time PCR. All data shown were mean values ± SD (error bar) from three replicates. *P* values were shown in the figures. **g** *NEAT1–1* pull-down assay use biotin-labeled *NEAT1–1* and *NEAT1–1* mutants (mut). The RNA and protein complexes were detected by western blot

The m6A site is usually on the 3′ untranslated region (UTR) of the mRNA like *NEAT1*’s neighbor gene *FRMD8* (Fig. S2a). To determine the real m6A site on the *NEAT1–1*, we search the m6A site via m6A RIP-seq data in cell. From the m6A RIP-seq data in 293 T cells by two independent m6A antibodies [38, 42], we found that there were 4 high peak on the *NEAT1–1* variant 1 (*NEAT1*–1), and low signals in other regions in *NEAT1* variant 2 (*NEAT1*–2) (Fig. 2a). In addition, we also found that RNA level of *NEAT1–1* was approximated 5 fold higher than *NEAT1–2* in P-18 cells (Fig. S2b). The m6A RIP data showed that m6A of *NEAT1–1* was approximate 10 fold higher than *NEAT1–2* in P-18 cells (Fig. S2b). Thus, we focused on the m6A of *NEAT1–1*. From 5′ to 3′ of *NEAT1*–1, we labeled the m6A sites from #1 to #4 (Figure S2c-f). Based on the conservative motif of m6A site, we found these sites inside the peaks and confirmed these sites by m6A RIP. The cross-linking immunoprecipitation (CLIP) polymerase chain reaction (PCR) assay showed that all four sites had the significantly higher m6A signals than the neighbor sites in prostate cancer P-18 cell (Fig. 2b).

Unlike the messenger RNA (mRNA) *FRMD8* (Fig. S2a), we found that the 4 m6A sites were distributed in whole region from 5′ to 3′ of *NEAT1–1*. It suggest that m6A on ncRNA may have a different function from mRNA. Next, we further want to know these m6A sites can affect the secondary structure of *NEAT1–1*, we used the RNA secondary structure prediction software ViennaRNA (https://rna.tbi.univie.ac.at) to predict the secondary structure of *NEAT1–1*. The prediction figure (Fig. S2c-f) showed that m6A sites #1, #2, #3 were inside the single strand, suggesting m6A modification can’t affect the double strand RNA interaction and secondary structure. The m6A site #4 in the double strand and m6A modification may block the double strand RNA interaction here (Fig. S2e). It was noticed that the site#4 region of *NEAT1–1* is a structural flexible region in published data [43, 44], suggesting that this region may take different secondary structure for different biological function. To know whether m6A mutation affects the half-life of *NEAT1–*1 RNA, we measured the half-life of *NEAT1–1* WT and site#4-mut in P-18 cell. The data showed that *NEAT1–1* WT half-life time was about 18 min in 293 T cell, consistent with other reports [45–47]. The half-life of *NEAT1–1* WT and site#4-mut were approximated 8 h and they were similar with each other (Fig. S2g). The data further showed that the half-life times of *NEAT1–1* in P-18 parental and METTL3 knocking-out (KO) cells had no significant difference (Fig. S2g). These data indicated that there were 4 definite m6A sites on *NEAT1–1* and site#4 may affect the secondary structure of *NEAT1–1*.

### *NEAT1–1* interacted with CYCLINL1 through m6A site #4

To find the molecular function for m6A on *NEAT1–1*, we generated *NEAT1–1* wild type (WT) and m6A deletion mutation plasmid for unbiased tandem affinity purification by biotin labeled probe and mass spectrometry (mass spec). Mass spec assay showed that *NEAT1–1* WT bound multiple proteins, including CYCLINL1, but not m6A mutation in primary bone metastatic P-18 cell (Fig. 2c and Table S2). Interestingly, we found multiple pathways related metastasis using Gene Ontology (GO) and Kyoto Encyclopedia of Genes and Genomes (KEGG) analyses, including the pathways were related to extracellular exosome, myelin sheath and lamin filament (Fig. 2d). To confirm the mass spec data, we used biotin labeled probe to target *NEAT1–1* to pull down interacted protein. The western blot data showed that only CYCLINL1 bound to *NEAT1–1*, but not CYCLINT1 and CYCLINC (Fig. 2e). To determine the m6A’s role in interaction with CYCLINL1, we knocked down the m6A writer METTL3 in P-18 cells (Fig. S3a). We observed that CYCLINL1 associated with *NEAT1–1* in P-18 cells by CLIP assay, but did not associate with *NEAT1–1* in METTL3 knocking-out P-18 cells (Fig. 2f). To further examine the m6A’s role in interaction with CYCLINL1, we performed the RNA pull-down assay with WT and site#4 m6A deletion mutated *NEAT1–1*. We revealed that only the #4 m6A site mutation blocked the binding to CYCLINL1, but not #1, #2 and #3 (Fig. 2g). Furthermore, Fig. 2f showed that site#4 m6A mediated the *NEAT1–1* interaction with CYCLINL1, but not ESR1, EZH2, and SUZ1. These data indicated that *NEAT1–1* interacted with CYCLINL1 through its #4 m6A site in prostate PDX primary cells.

### *NEAT1–1* is a bridge to connect CYCLINL1 and CDK19

**Fig. 3:**
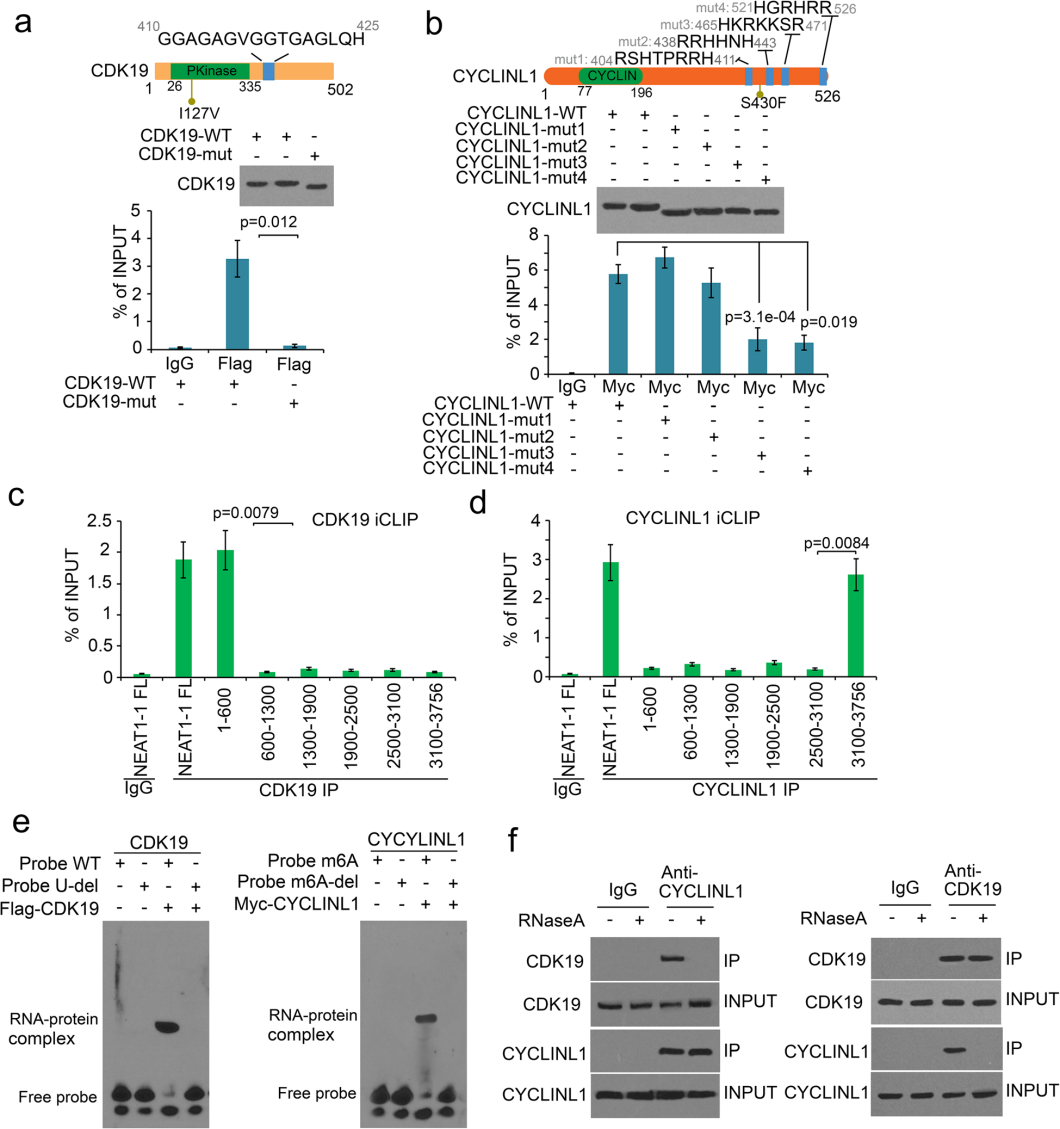
*NEAT1–1* interacted with CYCLINL1 and CDK19 through m6A site #4. **a** and **b** Upper, protein diagrams with RNA recognition motif (rrm) were shown. Lower, CLIP-qPCR analyswas of Flag or Myc binding at the *NEAT1* in P-18 out cells transfected with Flag-CDK19-WT, Flag-CDK19-mut (rrm deletion), Myc-CYCLINL1-WT or Myc-CYCLINL1-muts (rrms deletion). Nature mutations were shown in the diagrams. PKinase, protein kinase domain. All data shown were mean values ± SD (error bar) from three replicates. *P* values were shown in the figures. **c** and **d** CLIP-qPCR analyswas of CDK19 or CYCLINL1 binding at the *NEAT1–1* in P-18 cells with different primers targeting different regions. **e** RNA EMSA evaluation of CDK19 or CYCLINL1 binding of *NEAT1–1* RNA probes. Flag-CDK19 or Myc-CYCLINL1 recombinant proteins were produced using Quick coupled transcription/translation kit through T7 promoter in vitro. Flag-CDK19 or Myc-CYCLINL1 recombinant proteins were incubated with biotin-labeled in vitro transcribed *NEAT1–1* probes in the presence or absence of m6A modification (single nucleotide “A” deletion (3494 nt of *NEAT1–1*)), followed by PAGE and immune blotting with HRP-conjugated streptavidin. **f** Co-IP of endogenous CYCLINL1 or CDK19 with CDK19 or CYCLINL1 from P-18 cell lysate pre-treated with RNaseA in 37 °C for 30 min. The protein complexes were detected by western blot

Based on the mass spec data, we found that *NEAT1–1* bound to CYCLINL1 and Cyclin-dependent kinase (CDK)19 (Fig. 2c). To demonstrate direct or indirect binding between CYCLINL1 or CDK19 and *NEAT1–1*, we searched the potential RNA binding residues (RBR) in the CYCLINL1 and CDK19. We used RBRDetector (http://ibi.hzau.edu.cn/rbrdetector) and found a GxxGxG domain in CDK19 and four arginine-rich domains in CYCLINL1, which were potential RNA recognized motifs (RRMs) (Fig. 3a and b). Additionally, there was no nature mutation in RRMs and known domains in CDK19 and CYCLINL1 (Fig. 3a and b). GxxGxG RNA binding domain (G is glycine, x is any animo acid) is similar with the GxxGxG-domain in hnRNPU, which prefers to interact with U-rich RNA [48, 49]. The GxxGxG-domain is also a unique domain in CDK19, but not in its homolog CDK8 (Fig. S3b). GxxGxG domain deletion blocked the interaction with CDK19 and *NEAT1–1* by in vitro transcribed RNA in P-18 cells (Fig. 3a). The arginine-rich domain is one of RNA recognized domains for RNA binding [49, 50]. The two arginine-rich domains in the C-terminal of CYCLINL1 were required to interact with *NEAT1–1* in P-18 cell lysate (Fig. 3b). To further determine the *NEAT1–1* binding region in cell, we performed the interaction assay by individual-nucleotide resolution CLIP (iCLIP). The iCLIP assay showed that *NEAT1–1* bound to CDK19 inside 1–600 nt; and *NEAT1–1* bound to CYCLINL1 inside 3100–3756 nt (Fig. 3c and d). RNA electrophoretic mobility shift assay (EMSA) showed that CDK19 associated with the unique U-rich region (487–493 nt) in *NEAT1–1* 1–600 nt (Fig. 3e), which is consistent with CxxCxC-domain feature in hnRNPU binding [48]. RNA EMSA also showed that CYCLINL1 associated with m6A region inside *NEAT1–1*3100–3756 nt (Fig. 3e and S3c). Co-immunoprecipitation (Co-IP) assay demonstrated that destroy of RNA inhibited the interaction between CYCLINL1 and CDK19 (Fig. 3f). Taken together, these data indicated that *NEAT1–1* is a bridge to connect CYCLINL1 and CDK19.

Interestingly, CYCLINL1 is a bone specific expressed protein (Fig. S3d); CDK19 is a prostate specific expressed protein (Fig. S3d). We found that both of CYCLINL1 and CDK19 were highly expressed in bone metastatic prostate cancer tissues (Fig. S3e and f). These data suggest that the complex among CYCLINL1, CDK19 and *NEAT1–1* may be a specific complex in bone metastatic prostate cancer.

### *NEAT1–1* activates pol II Ser2 phosphorylation through m6A in vitro and in prostate cancer

**Fig. 4:**
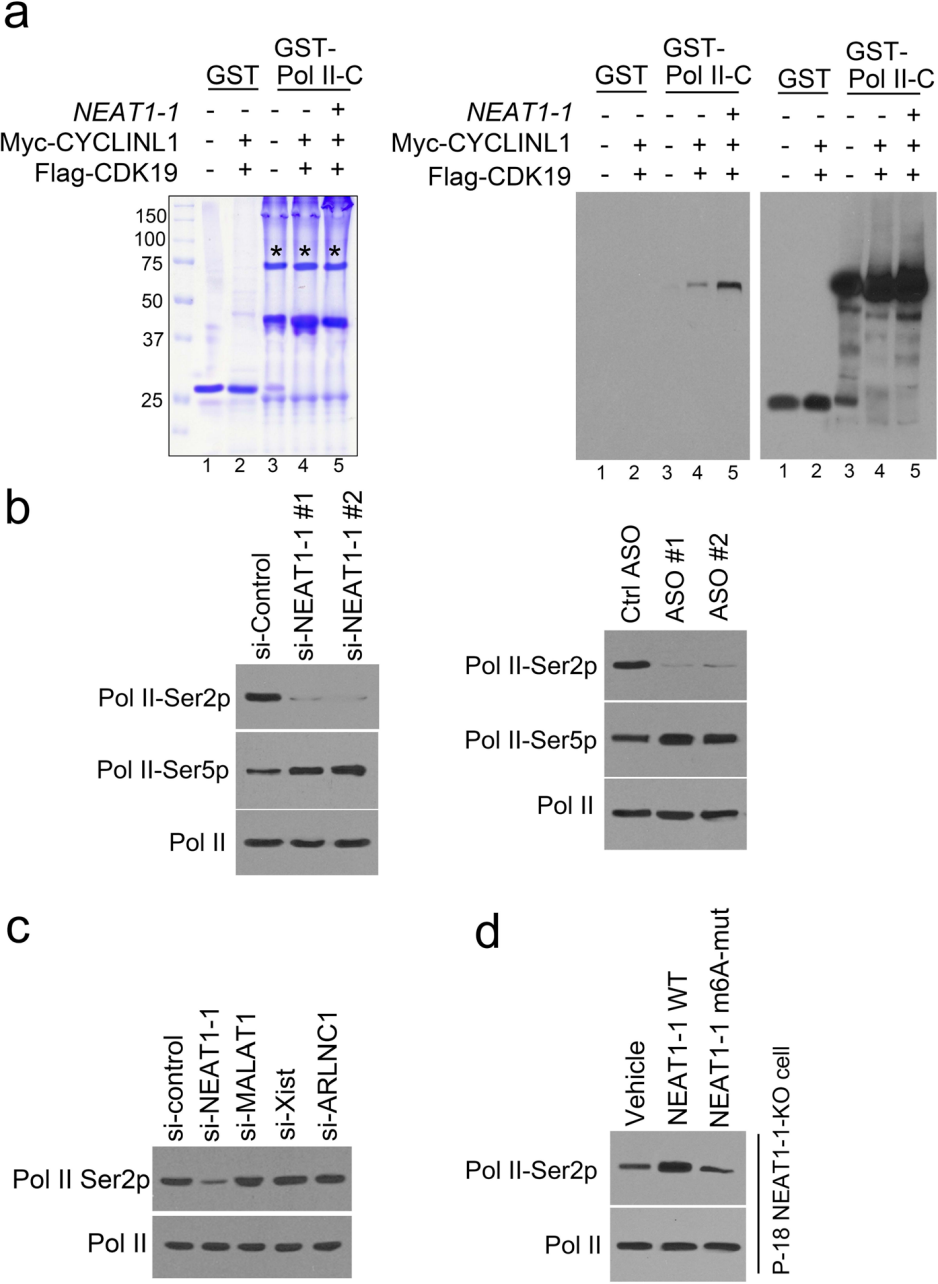
*NEAT1–1* activated Pol II Ser2 phosphorylation through m6A in vitro and in prostate cancer. **a** Left, Coomassie blue staining of GST and GST-Pol II-C terminal domain (GST-Pol II-C, 372 amino acids of the Pol II-C terminal domain) recombinant proteins used for kinase assay. A star indicate the GST and GST-Pol II-C recombinant protein bands. Right, determination of CTD kinase (CDK19) activity. 1 μg GST or GST-Pol II-C terminal fusion proteins (in all lanes) and 2 μg *NEAT1–1* RNA (lane 5) were incubated with CDK19 (lane 2,4,5) and CYCLINL1 produced from Quick coupled transcription/translation kit. After extensive washing, GST-beads were collected and subject to SDS/PAGE and western blot with Pol II Ser-2p antibodies. **b** Expression of Pol II Ser-2, Sre-5 phosphorylations and total Pol II proteins were measured by western blot in P-18 cells infected with control or *NEAT1*-specific siRNAs or in transfected with control or *PSA* eRNA-specific ASOs. **c** Expression of Pol II Ser-2 phosphorylations and total Pol II proteins were measured by western blot in P-18 cells with control siRNAs and siRNA pool of *NEAT1, MALAT1, Xwast* or *ARLNC1*. **d** Expression of Pol II Ser-2 phosphorylations and total Pol II proteins were measured by western blot in P-18 *NEAT1–1*-KO cells infected with control or *NEAT1–1*-overexpressed plasmids or *NEAT1–1*-m6A-mutants plasmids

CDK19 is a homolog of CDK8, and they are cyclin-dependent kinase that regulates RNA Polymerase II (RNPII) to mediate gene expression [51–53]. CDK8 with its partner CYCLINC phosphorylates the CTD of RNPII in vitro and in vivo [51]. However, the molecular detail for CDK19 regulation is limited. In this study, we found CDK19 can be pulled down by *NEAT1–1* in P-18 (Fig. 2c). To examine the activity of CDK19, *NEAT1–1* and CYCLINL1 complex, we consequently translated the CDK19 or CYCLINL1 in vitro using TNT quick coupled transcription/translation system kit and specific magnetic beads. Before the kinase assay in vitro, we determined the m6A of *NEAT1–1* in vitro. The dot blot assay showed that *NEAT1–1* RNA can be methylated in P-18 cell, but not in METTL3-KO cells (Fig. S4a). Then we purified the *NEAT1–1* from these cells by biotin labeled probes. In vitro RNPII phosphorylation assay showed that adding *NEAT1–1* activated the RNPII phosphorylation in Ser-2 (Fig. 4a and S4a). Next, we found that *NEAT1–1* binding domain-deletion in CYCLINL1 blocked the RNPII Ser2 phosphorylation induced by CDK19 and CYCLINL1 in vitro (Fig. S4b). However, the kinase activity of complex formed by CDK19 and CYCLINC was not blocked by *NEAT1–1* binding domain-deletion (Fig. S4b). These data suggest that the kinase activity of complex formed by CDK19 and CYCLINL1 required *NEAT1–1* interaction. It was noticed that most of *NEAT1–1* are located in nucleus [54–56]. We checked *NEAT1–1’*s location in P-18 cells, the data showed that transfected *NEAT1–1* was located in nucleus in P-18 cell (Fig. S4c), suggesting the *NEAT1–1* may take nuclear function in prostate cancer cell. We demonstrated that knocking out *NEAT1–1* in P-18 by siRNAs and ASOs deterred the RNPII phosphorylation in Ser-2, but not Ser-5 (Fig. 4b). In addition, we found that RNPII Ser2 phosphorylation was downregulated by depletion of *NEAT1–1*, but not depletion of *MALAT1*, *Xist* or *ARLNC1* (Fig. 4c and S4d). To further determine the exact the m6A site, we transfected the #4 site-mutation (3494A deletion) *NEAT1–1* into P-18 cells with *NEAT1–1* homogeneous knocking out (KO) by CRISPR system. We confirmed that transcribed *NEAT1–1* increased the m6A-modified *NEAT1–1* level in P-18 *NEAT1–1*-KO cells (Fig. S4e) and also confirmed the homogeneous *NEAT1–1* KO efficacy and ruled out the off-target effect (Fig. S5a-c). We found that *NEAT1–1* WT increased the RNPII Ser-2 phosphorylation, but #4 mutation failed to do it (Fig. 4d). These data indicated that *NEAT1–1* activated RNPII Ser-2 phosphorylation through #4 m6A site on *NEAT1–1*.

### *NEAT1–1* recruites CYCLINL1 and CDK19 on *RUNX2* promoter via m6A site #3

**Fig. 5:**
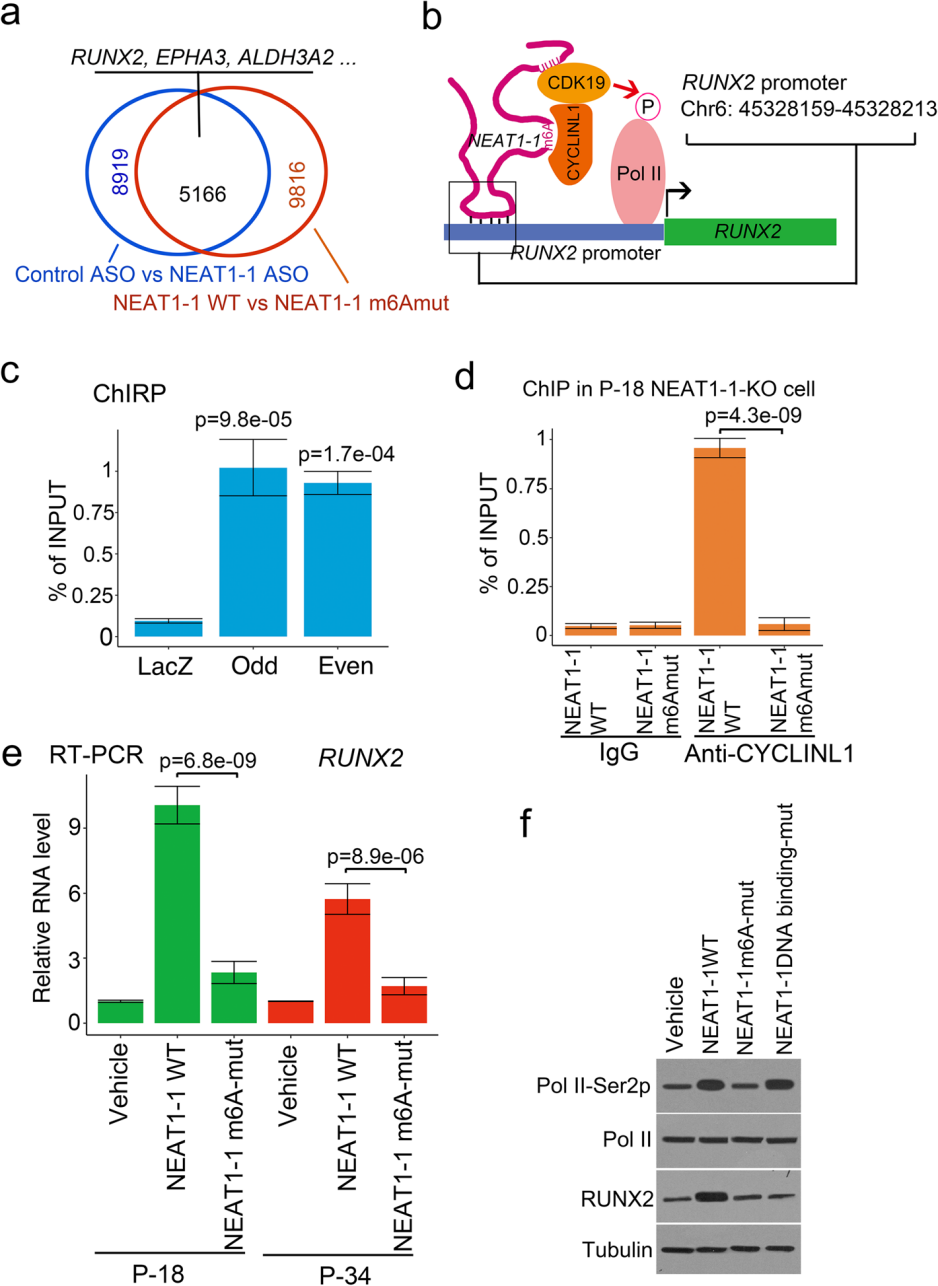
*NEAT1–1* recruited CYCLINL1 and CDK19 on RUNX2 promoter via m6A site #4. **a** Venn diagram showing that genes mediated by *NEAT1–1* knocking down overlapped with genes mediated by *NEAT1–1* site#4 m6A deletion (nt3494A deletion) in P-18 cells (*P* = 9.3e-10, permutation test). **b** A diagram of CDK19 and CYCLINL1 interaction through *NEAT1* on promoter of target gene. **c** ChIRP assay using biotin-labeled LacZ or *NEAT1–1*-specific DNA probes and streptavidin beads. Pulldown DNA was analyzed by real-time PCR. All data shown were mean values ± SD (error bar) from three replicates. *P* values were shown in the figures. **d** CHART-qPCR analyswas of CYCLINL1 binding at the *NEAT1–1* WT and m6A-mut in P-18 *NEAT1*-KO cells. Immunoprecipitated RNAs were detected by real-time PCR. All data shown were mean values ± SD (error bar) from three replicates. m6A-mut was site#4 m6A deletion in *NEAT-1*. *P* values were shown in the figures. **e** RUNX2 expressions were measured by qRT-PCR in P-18 and P-34 primary cells. Means and standard deviations (error bar) were determined from three replicates. Error bars represent mean ± SD for triplicate experiments. m6A-mut was site#4 m6A deletion in *NEAT-1*. *P* values were shown in the figures. **f** Expression of RUNX2, Tubulin, Pol II Ser-2 phosphorylations and total Pol II proteins were measured by western blot in P-18 *NEAT1–1*-KO cells infected with control or *NEAT1–1*-WT, *NEAT1–1*-m6A-mutant (nt3494A deletion) or *NEAT1–1* promoter-binding mutant (nt874–899 deletion)

To search the target genes of CYCLINL1/CDK19/*NEAT1–1* complex, we performed RNA-seq to find the regulated genes in two groups. One group was ASO control vs. *NEAT1–1* KD group; another group was P-18 *NEAT1–1* KO cells transfected with *NEAT1–1* WT vs. *NEAT1–1* site#4-m6A deletion. The venn diagram data showed that 5166 genes were the target genes that can be regulated by both depletion of *NEAT1–1* and deletion of *NEAT1–1* site#4 m6A (Fig. 5a, Table S3 and Table S4). Some target genes were bone metastasis related genes, such as *RUNX2, EPHA3* and *ALDH3A2* [57–59]. We confirmed the RNA levels’ changes in *NEAT1–1* WT and site#4-m6A deletion cells by RT-PCR (Fig. S5d). RUNX2 is a central driver in bone metastatic prostate cancer [58, 60–62]. To further search the target gene of complex of *NEAT1–1*, CYCLINL1 and CDK19, we hypothesis that the complex select its target through RNA-DNA interaction. We predicted the potential target gene by DNATriplex software (http://lncrna.smu.edu.cn/show/DNATriplex). The prediction data showed that *NEAT1–1* may interacted with the promoter of *RUNX2*, which is vital marker and driver in bone metastatic prostate cancer (Fig. 5b). To confirm this prediction, we performed the chromatin isolation by RNA purification (ChIRP) in P-18. The ChIRP data revealed that *NEAT1–1* interacted with the promoter of *RUNX2* in − 200 nt ~ − 50 nt region (Fig. 5c), consistent with software prediction result. To know whether *NEAT1–1* recruited CYCLINL1 or CDK19 onto promoter of *RUNX2*, we tested the binding of CYCLINL1 by chromatin immunoprecipitation (ChIP) assay. ChIP data showed that depletion of *NEAT1–1* by CRISPR system and rescued with *NEAT1–1* #4 m6A-mut blocked the association of CYCLINL1 with promoter of *RUNX2* (Fig. 5d). Furthermore, overexpression of *NEAT1–1* WT increased the *RUNX2* RNA and protein levels, but *NEAT1–1* m6A #4-mutation failed to upregulate *RUNX2* RNA in P-18 cells and P-34 cells and protein levels in P-18 cells (Fig. 5e and f). Taken together, these data #4 m6A site facilities *NEAT1–1* recruit CYCLINL1 and CDK9 onto promoter of RUNX2 through RNA-DNA interaction.

### *NEAT1–1* enhanced prostate PDX growth through m6A

**Fig. 6:**
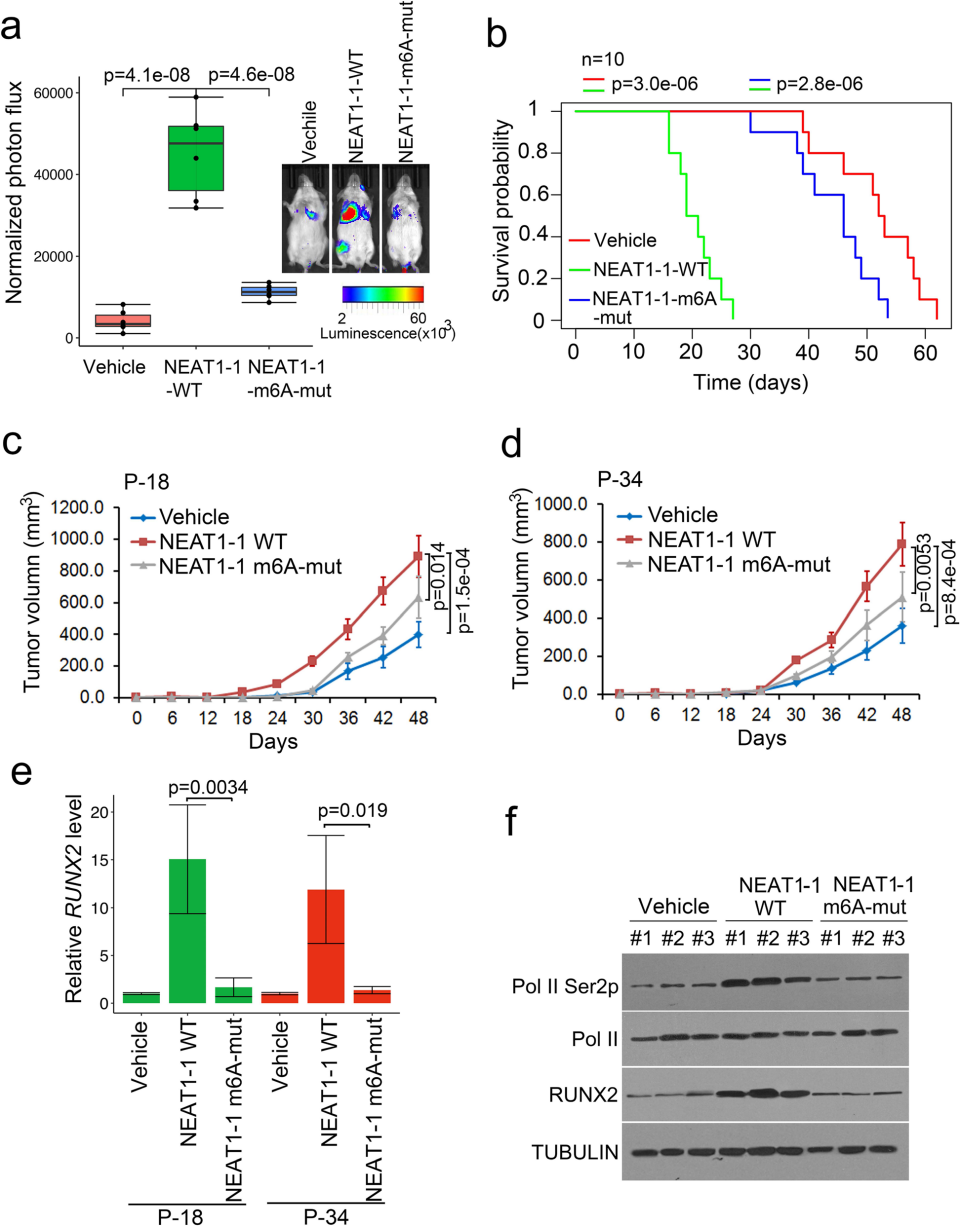
*NEAT1–1* enhanced prostate PDX metastaswas and growth through m6A. **a** Luciferase-expressing P-18 cells (3 × 10^6^) transfected with lentivirus were injected through tail vein into NSG mice (*n* = 10 each group). The mice were subjected to bioluminescent imaging and bioluminescent signals were quantified. Bioluminescent flux (photons/s/sr/cm^2^) was determined for lesions in mice, the ends of the box were the upper and lower quartiles and the box spans the interqurtile range; the median was marked by a vertical line inside the box; the whiskers were the two lines outside the box that extend to the highest and lowest observations. *P* values were shown in the figures. Representative images from 10 different samples were shown. **b** Kaplan-Meier survival analyswas of the mice data for the survival time in three groups. Three groups were indicated in the figure. **c** and **d** Effect of m6A of *NEAT1* on growth of bone metastatic prostate cancer xenografts. 5 × 10^6^ P-18 and P-34 cells were injected into NSG mice (*n* = 6 each group). The tumor growth was measured every 6 days for 48 days, and the data were shown in the bottom panel. Data shown as means ± SD (n = 6). Statwastical significance was determined by two-tail Student’s *t*-test. *P* values were shown in the figures. **e** and **f** RUNX2 expressions were measured by qRT-PCR and western blot in P-18 and P-34 xenograft tissues. Means and standard deviations (error bar) were determined from three replicates. Error bars represent mean ± SD for triplicate experiments. *P* values were shown in the figures. Expression of RUNX2, Tubulin, Pol II Ser-2 phosphorylations and total Pol II proteins were measured by western blot in P-18 three random xenograft tissues

To test the m6A of *NEAT1–1* function in vivo, we used the xenograft model to determine what extent m6A regulation of *NEAT1–1* contributes to prostate PDXs growth and metastasis. The mice tail vein injection data showed that overexpression of *NEAT1–1* WT decrease the mice survival span, and also increase the metastatic probability to pelvis bone and/or lung (Fig. 6a, b and S6a). Furthermore, *NEAT1–1* WT promoted flank tumor growth of P-18 and P-34 PDXs, but not *NEAT1–1* #4 m6A-mutant (Fig. 6c and d). The RT-PCR data in each xenograft sample showed that *NEAT1–1* WT and m6A-mut groups had higher levels of *NEAT1–1* RNAs than vehicle groups of P-18 cell and P-34 cell xenografts. However the level of *NEAT1–1* was no significant difference among the xenografts in *NEAT1–1* WT and m6A-mut overexpression groups (Fig. S6b). *RUNX2* RNA levels were significantly higher in *NEAT1–1* WT-overexpressed flank tumors than m6A-mut overexpressed tumors (Fig. 6e). Protein levels of RUNX2 were also higher in *NEAT1–1* WT-overexpressed flank tumors than m6A-mut overexpresed tumors (Fig. 6f). The data indicated that *NEAT1–1* WT upregulated the phosphorylation of RNPII Ser2 and *RUNX2* RNA and protein levels compared to vehicle group, but the m6A mutation group failed to do these (Fig. 6e and f). Therefore, the data demonstrated that m6A of *NEAT1–1* elevated the tumor growth and metastasis in mice PDX model.

## Discussion

m6A in mammalian was identified as a highly abundant modification of mRNA several decades ago [7–9]. The writers, erasers and readers of m6A can add, remove and recognize the m6A site in mRNA and do the function including: nuclear transport, splicing, stability and translation [10–12, 14]. In long noncoding RNA, m6A was found on metastasis-associated lung adenocarcinoma transcript-1 (*MALAT1*) [63] and X inactive-specific transcript (*Xist*) [64]. In this study, we found a new m6A modified long noncoding RNA - *NEAT1–1*. The m6A level of *NEAT1–1* was positively related to prostate cancer progression, bone metastasis and negatively related to patients’ survival (Fig. 1). It indicated that m6A in *NEAT1–1* may exhibit oncogenic function in cancer progression. m6A in *MALAT1* associates with HNRNPC to affect target gene expression [63] and m6A in *Xist* binds to YTHDC1 to mediate the transcript silencing [64]. Here, we found a new m6A reader - CYCLINL1. CYCLINL1 is a bone marrow specific protein [65]. Interestingly, we found that CYCLINL1 also expressed in prostate cultured primary cell and PDXs from bone metastatic patients. CYCLINL1 bound to *NEAT1–1* m6A site with its R-rich C-terminal domain. High m6A level *NEAT1–1* activated CYCLINL1 and formed a new complex with CDK19 which was specific high expressed in prostate tissues. Thus, *NEAT1–1* induced the activity of CYCLINL1/CDK19 complex, and recruited it onto *RUNX2* promoter through RNA/DNA hybridization. Therefore, we identified the new m6A function in long noncoding RNA and pushed prostate cancer progression going ahead.

Prostate cancer is the second-most common cause of cancer death in men of US [1, 2]. The main mortality cause of prostate cancer is metastasis and about 80% of the metastatic prostate cancer cells spread to bones [3, 4]. The reason why prostate cancer settling in bone “soil” is limited to know. In mechanism, how prostate cancer express bone related genes and synchronize itself to be adapted to bone environment is also an interesting question. There were several key proteins found in in bone and bone metastatic prostate cancer, including RUNX2 [58, 60, 61]. Interestingly, CYCLINL1 is a bone specific expressed protein; CDK19 is a prostate specific expressed protein (Fig. S3). We found that both of CYCLINL1 and CDK19 were highly expressed in bone metastatic prostate cancer tissues. These data suggest that the complex from CYCLINL1, CDK19 and *NEAT1–1* might be a specific complex in bone metastatic prostate cancer. We also found that the high activation of CYCLINL1 was related to high level of m6A in *NEAT1–1*. *NEAT1–1* activated the CYCLINL1 through the m6A site interaction with CYCLINL1. Depletion of *NEAT1–1* or mutation in the m6A of *NEAT1–1* inhibited the CYCLINL1 activity and *RUNX2* RNA levels, which was the downstream target of CYCLINL1 and CDK19 in cell and xenograft (Fig. 6). With the RUNX2 and related pathway, bone metastatic prostate cancer exhibited the similar features with bone and can be survival in the environment of bone.

Taken together, we found the *NEAT1–1* m6A facilitated the oncogenic function of new complex CYCLINL1/CDK19 in bone metastatic prostate PDXs. The findings indicate that ncRNA m6A have vital role in regulating prostate cancer progression and may be novel target for cancer therapy and diagnosis.

## Conclusions

In conclusion, we identified the novel m6A function in ncRNA that played an oncogenic role in bone metastatic prostate cancer and was correlated with poor prognosis. Further experiments demonstrated that *NEAT1–1* m6A facilitated the oncogenic function of new complex CYCLINL1/CDK19 for Pol II Ser2 phosphorylation. Our results revealed that ncRNA m6A had vital role in regulating prostate cancer progression and may be novel target for cancer therapy and diagnosis. The regulatory network involving the new complex CYCLINL1/CDK19/*NEAT1–1* might provide new insight into the potential mechanism of the pathogenesis and development of bone metastatic prostate cancer.

## Supplementary Information

**Additional file 1: Supplemental Fig. 1**. m6A of *NEAT1–1* was elevated in prostate cancer and was a negative prognostic factor for patients. (a) Box and whisker plot showing *NEAT1–1* RNA signals upregulated in prostate cancer tissues compwered to normal tissues in TCGA data set. Data from GEPIA. (b) Box and whisker plot showing *NEAT1* m6A signals upregulated in bone metastatic prostate cancer tissues. Analyswas of Tianjin Medical University data sets with fresh samples for levels of m6A and *NEAT1* RNA were based on the m6A-RIP and RT-PCR. *n* = 30 each group. *P* values were shown in the figures. (c) Kaplan-Meier survival analyswas of the TCGA data set for the relationship between the levels of NEAT, expression of *NEAT1–1* and survival time in prostate cancers. Data from GEPIA. **Supplemental Fig. 2**. m6A sites and secondary structure in *NEAT1*. (a) m6A RIP-seq analyswas of m6A sites of *FRMD8* by two independent antibodies. The m6A profiles of FRMD8 were shown in genome browser. (b) *NEAT1–1* and *NEAT1–2* expressions were measured by qRT-PCR in P-18 primary cells. M6A levels of *NEAT1–1* and *NEAT1–2* were measured by m6A-RIP-PCR in P-18 primary cells. Means and standard deviations (error bar) were determined from three replicates. Error bars represent mean ± SD for triplicate experiments. *P* values were shown in the figures. (c-f) Secondary structure of *NEAT1–1* predicted by https://rna.tbi.univie.ac.at. m6A putative motif and sequences were shown in each figures. (g) *NEAT1–1* expressions were measured by qRT-PCR in 293 T and P-18 primary cells. Transfected *NEAT1–1* expressions were measured by qRT-PCR using primers targeting *NEAT1–1* and plasmid in P-18 primary cells. Means and standard deviations (error bar) were determined from three replicates. Error bars represent mean ± SD for triplicate experiments. *P* values were shown in the figures. **Supplemental Fig. 3**. CYCLINL1 and CDK19 in tissues. (a) Expression of METTL3 and Vinculin proteins were measured by western blot in P-18 METTL3-KO cells infected with control or METTL3 KO CRWASPR-Cas9 plasmids. (b) Amino acid sequence comparwason of CDK8 and CDK19. (c) Dot blot of m6A and biotin to testing *NEAT1–1* WT and m6A-deletion probes using anti-m6A and anti-biotin antibodies. 0.5 μg RNA for each dot. (d) CYCLINL1 and CDK19 RNA levels in normal human tissues. Data from GTEx. (e and f) Box and whisker plot showing CYCLINL1 and CDK19 signals upregulated in bone metastatic prostate cancer tissues. Analyswas of Tianjin Medical University data sets for levels of CYCLINL1 and CDK19 RNA were based on the RT-PCR. n = 30 each group. **Supplemental Fig. 4.** *NEAT1–1* interacted with CYCLINL1 and CDK19. (a) Left panel, dot blot of m6A to testing NEAT1–1 full length RNA using anti-m6A. 0.5 μg RNA for each dot. Methylene blue was used to measure input loading. Right panel, 1 μg GST or GST-Pol II-C terminal fusion proteins and 2 μg *NEAT1–1* RNA were incubated with CDK19 and CYCLINL1 produced from Quick coupled transcription/translation kit. After extensive washing, GST-beads were collected and subject to SDS/PAGE and western blot with Pol II Ser-2p, total Pol II and GST antibodies. (b) GST or GST-Pol II-C terminal fusion proteins and 2 μg *NEAT1–1* RNA were incubated with CDK19 and CYCLINL1 WT or mutated proteins produced from Quick coupled transcription/translation kit from T7 promoter. After extensive washing, GST-beads were collected and subject to SDS/PAGE and western blot with Flag, Myc, Pol II Ser-2p, total Pol II and GST antibodies. (c) *NEAT1–1* RNA (green) and *AR* RNA (red) were stained using probes with FITC or TEXAS-RED dye. Scale bar was 10 μm. (d) *NEAT1–1, MALAT1, Xist* and *ARLNC1* expressions were measured by qRT-PCR in P-18 primary cells. Means and standard deviations (error bar) were determined from three replicates. Error bars represent mean ± SD for triplicate experiments. *P* values were shown in the figures. (e) *NEAT1–1* site#4 m6A levels were measured by m6A RIP PCR using primers targeting site#4 in P-18 primary cells. Means and standard deviations (error bar) were determined from three replicates. Error bars represent mean ± SD for triplicate experiments. *P* values were shown in the figures. **Supplemental Fig. 5**. Sanger sequence showed *NEAT1–1* knocking out in P-18 cells. (a) gRNAs sequences were shown in the two sides of *NEAT1–1* RNA in genome. (b) *NEAT1–1* full length and knocking out PCR products were amplified using primers outside the *NEAT1–1*. (c) gRNAs sequences were shown in the possible target in chromatin 22. (d) Target genes’ expressions were measured by qRT-PCR in P-18 *NEAT1–1* KO cells transfected with *NEAT1–1* WT and *NEAT1–1* m6A-mut. Means and standard deviations (error bar) were determined from three replicates. Error bars represent mean ± SD for triplicate experiments. *P* values were shown in the figures. **Supplemental Fig. 6**. X-ray for detection of mice bone. (a) Mice were scanned using a microcomputed x-ray system. Representative images from 10 different samples were shown. (b) *NEAT1–1* expressions were measured by qRT-PCR in P-18 and P-34 xenografts. Means and standard deviations (error bar) were determined from six replicates (*n* = 6). Error bars represent mean ± SD for triplicate experiments. NS, no significance.

**Additional file 2.**

**Additional file 3.**

**Additional file 4.**

**Additional file 5.**

## Data Availability

All data that support the findings of this study are available from the corresponding authors upon reasonable request.

## Abbreviations

m6A: N6-methyladenosine
*NEAT1*: Nuclear-enriched abundant transcript 1
ncRNA: noncoding RNA
CDK: Cyclin-dependent kinase
CLIP: Cross-linking immunoprecipitation
PCR: Polymerase chain reaction
qPCR: Quantitative real-time PCR
RBR: RNA binding residues
RIP: RNA immunoprecipitation
3′ UTR: 3′-untranslated region
