# Supplementary figures and images for: Long non-coding RNA NEAT1 promotes bone metastasis of prostate cancer through N6-methyladenosine
Source: Mol Cancer. 2020 Dec 12;19:171. doi: 10.1186/s12943-020-01293-4 (PMC7733260; doi:10.1186/s12943-020-01293-4)

# Figure S1

**a**

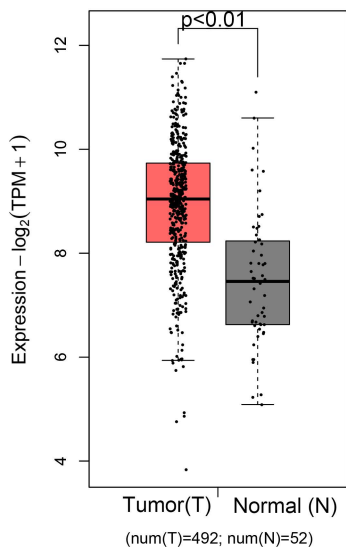

**c**

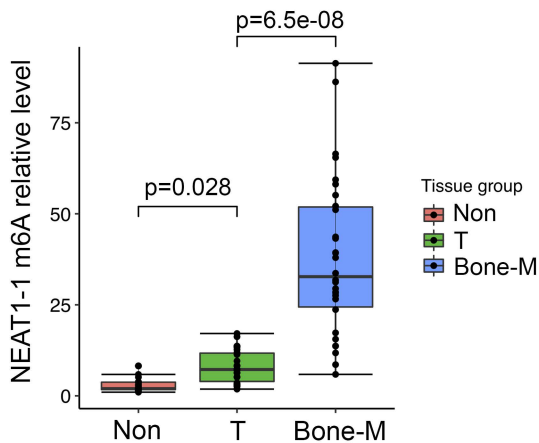

**b**

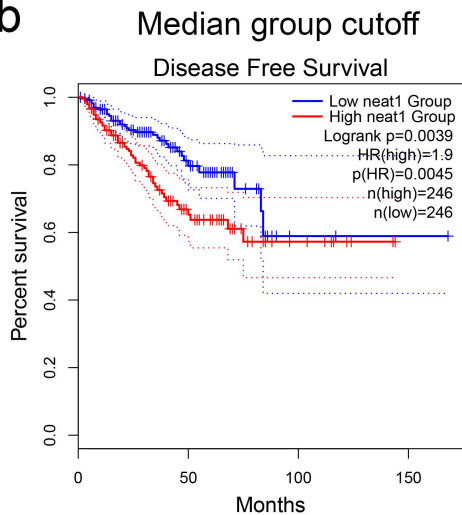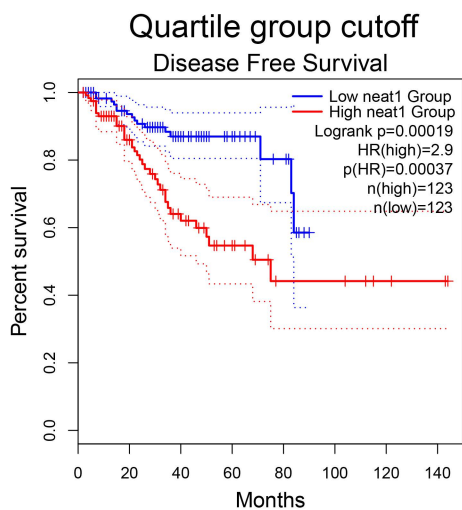

Figure S2

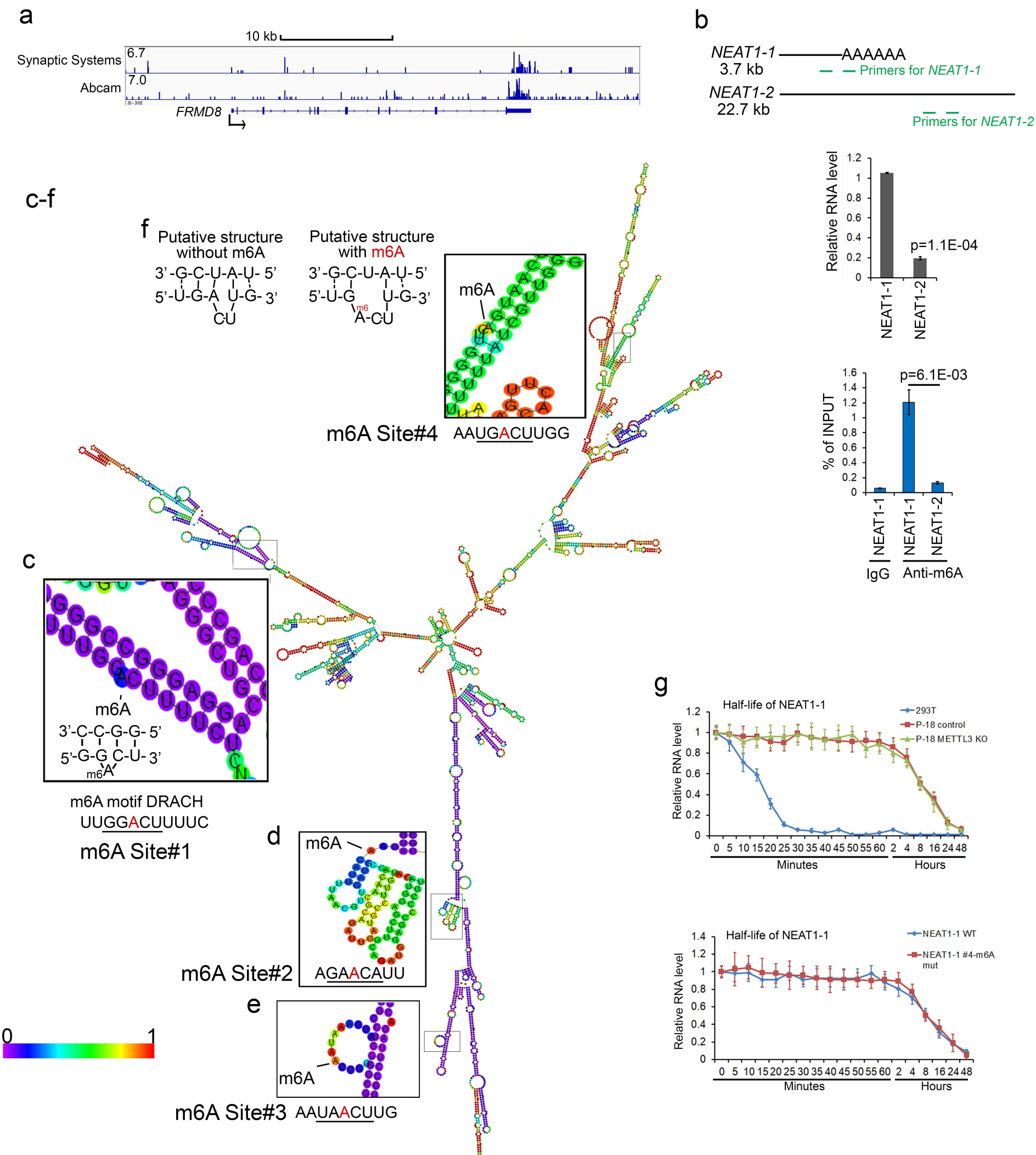

a

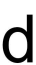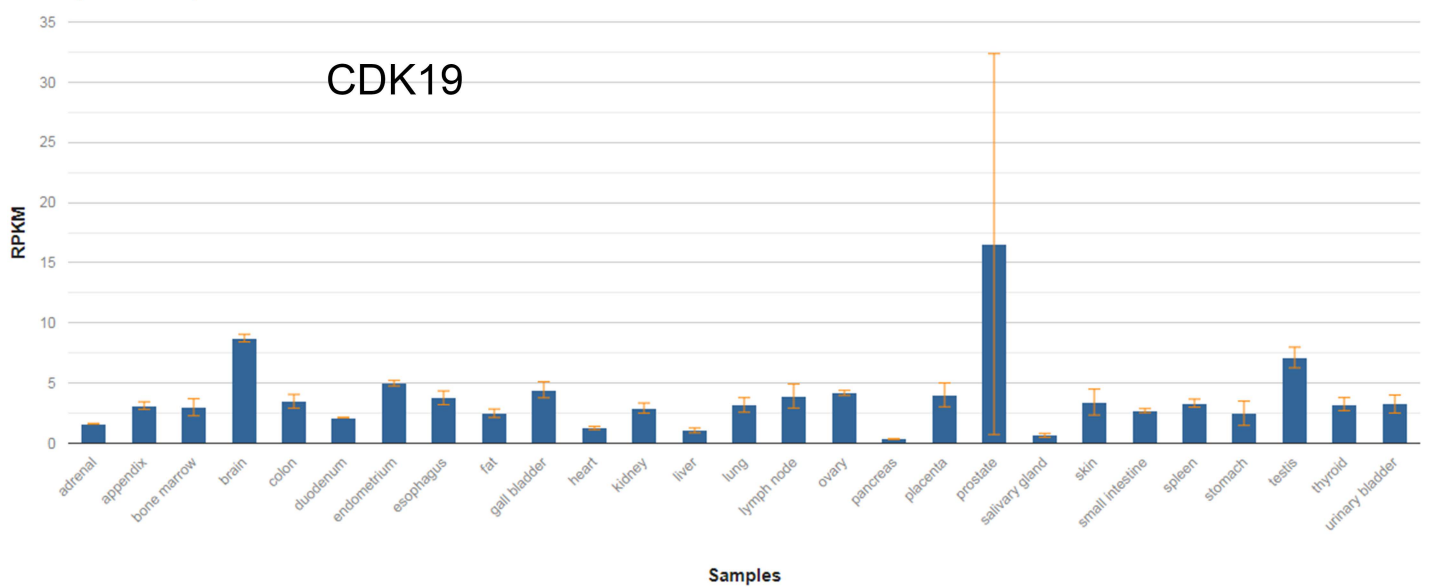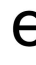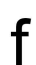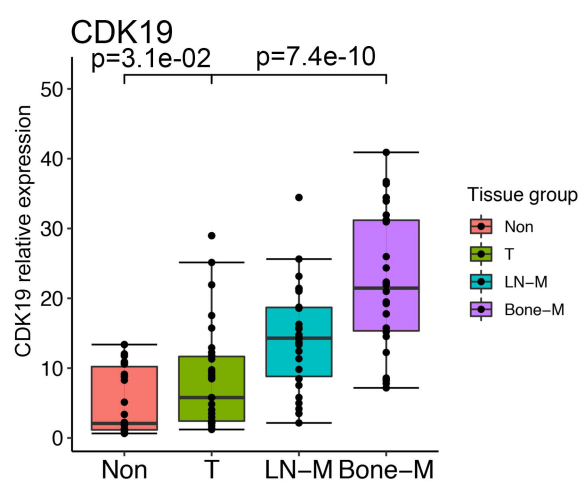

Figure S4

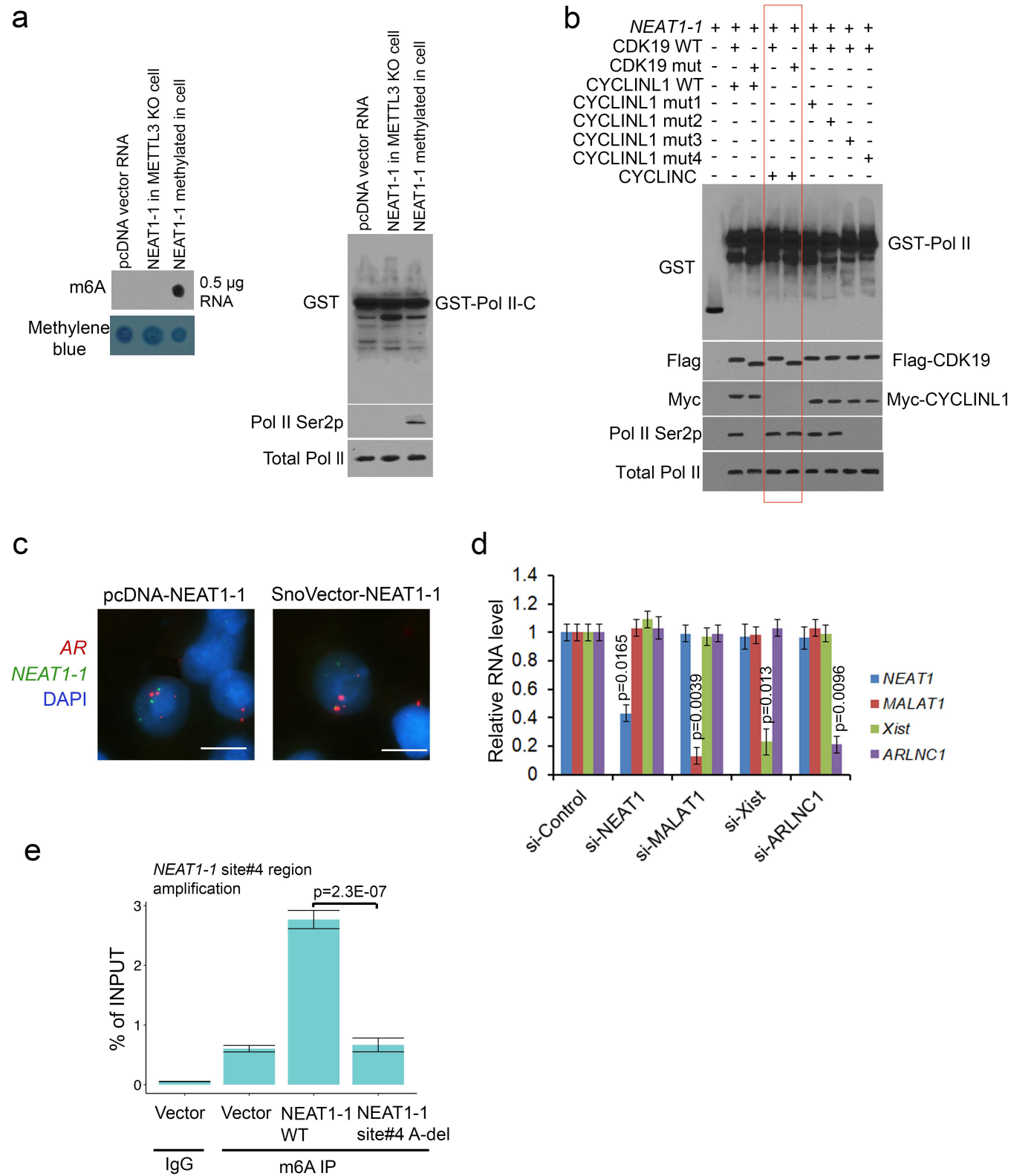

Figure S5

a

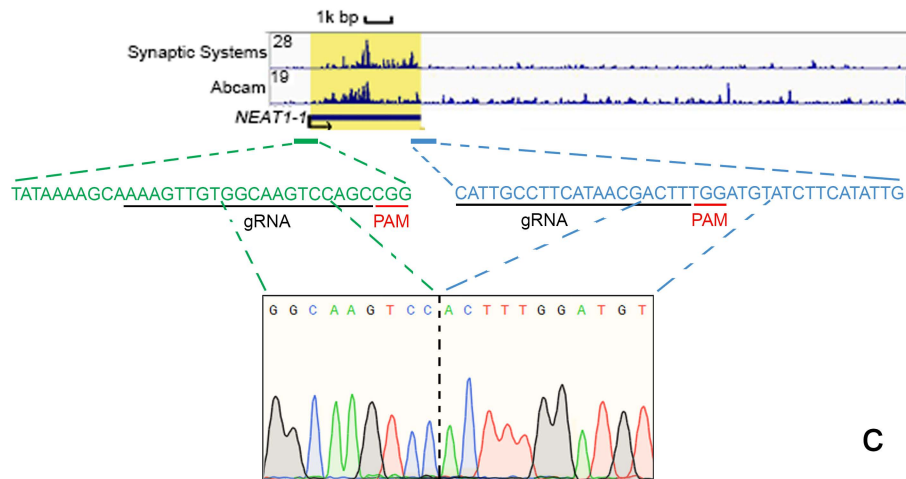

b

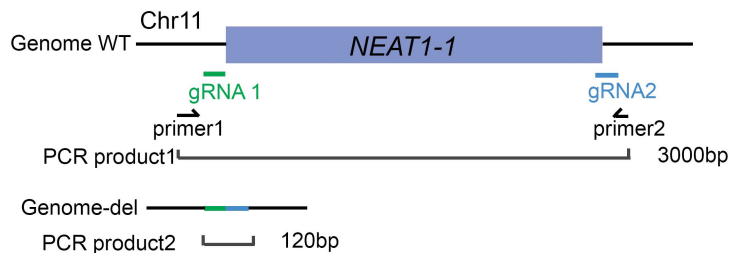

c

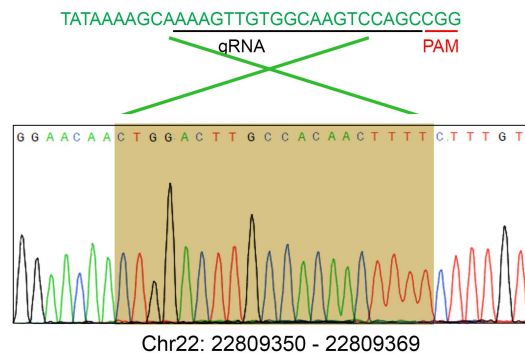

d

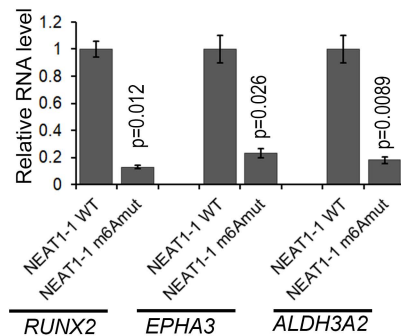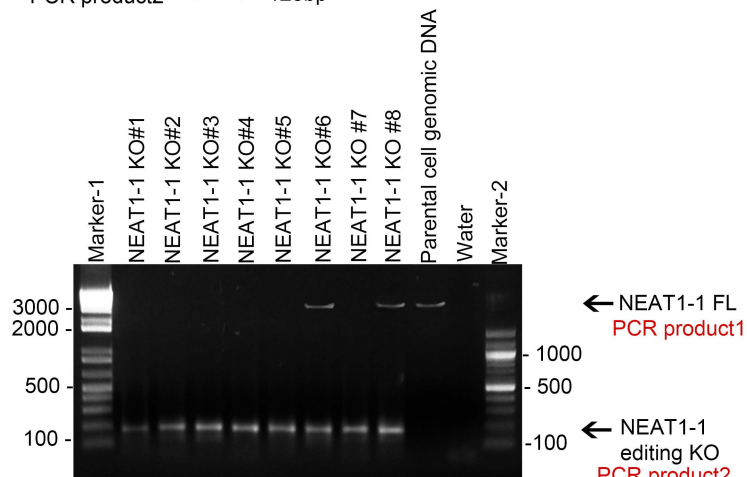

Figure S6

a

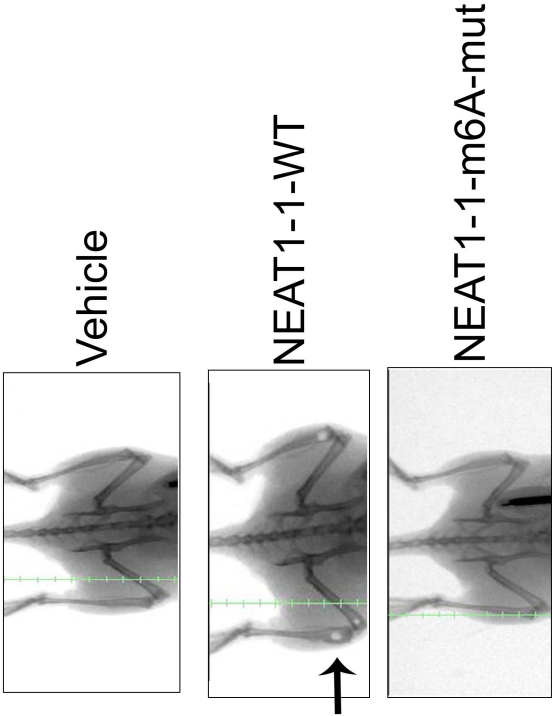

b

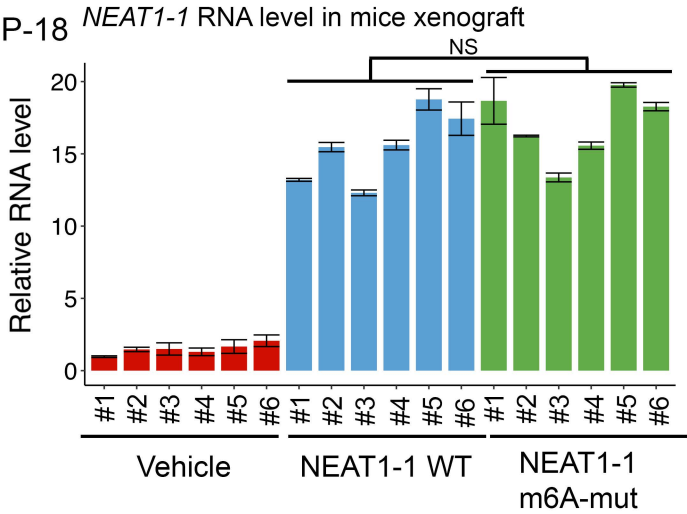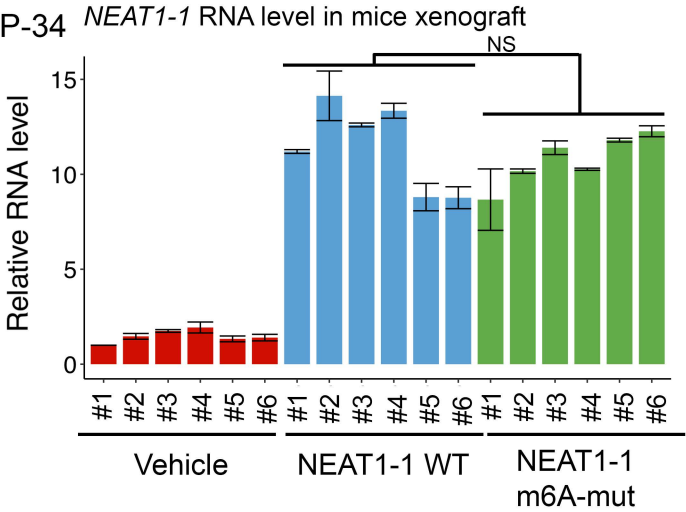

Supplement: Supplementary file 1 — Additional file 1: Supplemental Fig. 1. m6A of NEAT1–1 was elevated in prostate cancer and was a negative prognostic factor for patients. (a) Box and whisker plot showing NEAT1–1 RNA signals upregulated in prostate cancer tissues compwered to normal tissues in TCGA data set. Data from GEPIA. (b) Box and whisker plot showing NEAT1 m6A signals upregulated in bone metastatic prostate cancer tissues. Analyswas of Tianjin Medical University data sets with fresh samples for levels of m6A and NEAT1 RNA were based on the m6A-RIP and RT-PCR. n = 30 each group. P values were shown in the figures. (c) Kaplan-Meier survival analyswas of the TCGA data set for the relationship between the levels of NEAT, expression of NEAT1–1 and survival time in prostate cancers. Data from GEPIA. Supplemental Fig. 2. m6A sites and secondary structure in NEAT1. (a) m6A RIP-seq analyswas of m6A sites of FRMD8 by two independent antibodies. The m6A profiles of FRMD8 were shown in genome browser. (b) NEAT1–1 and NEAT1–2 expressions were measured by qRT-PCR in P-18 primary cells. M6A levels of NEAT1–1 and NEAT1–2 were measured by m6A-RIP-PCR in P-18 primary cells. Means and standard deviations (error bar) were determined from three replicates. Error bars represent mean ± SD for triplicate experiments. P values were shown in the figures. (c-f) Secondary structure of NEAT1–1 predicted by https://rna.tbi.univie.ac.at. m6A putative motif and sequences were shown in each figures. (g) NEAT1–1 expressions were measured by qRT-PCR in 293 T and P-18 primary cells. Transfected NEAT1–1 expressions were measured by qRT-PCR using primers targeting NEAT1–1 and plasmid in P-18 primary cells. Means and standard deviations (error bar) were determined from three replicates. Error bars represent mean ± SD for triplicate experiments. P values were shown in the figures. Supplemental Fig. 3. CYCLINL1 and CDK19 in tissues. (a) Expression of METTL3 and Vinculin proteins were measured by western blot in P-18 METTL3-KO cells [file 12943_2020_1293_MOESM1_ESM.pdf]
